# Dynamics of bacterial insertion sequences: can transposition bursts help the elements persist?

**DOI:** 10.1186/s12862-015-0560-5

**Published:** 2015-12-21

**Authors:** Yue Wu, Richard Z. Aandahl, Mark M. Tanaka

**Affiliations:** School of Biotechnology & Biomolecular Sciences, University of New South Wales, Sydney, 2052 NSW Australia; Evolution & Ecology Research Centre, University of New South Wales, Sydney, 2052 NSW Australia; Present address: Telethon Kids Institute, University of Western Australia, Perth, 6008 WA Australia

**Keywords:** Transposable elements, Mobile DNA, Bacterial evolution, Stress response, Transposition burst, Regulatory mechanism, Simulation model, Adaptation

## Abstract

**Background:**

Currently there is no satisfactory explanation for why bacterial insertion sequences (ISs) widely occur across prokaryotes despite being mostly harmful to their host genomes. Rates of horizontal gene transfer are likely to be too low to maintain ISs within a population. IS-induced beneficial mutations may be important for both prevalence of ISs and microbial adaptation to changing environments but may be too rare to sustain IS elements in the long run. Environmental stress can induce elevated rates of IS transposition activities; such episodes are known as ‘transposition bursts’. By examining how selective forces and transposition events interact to influence IS dynamics, this study asks whether transposition bursts can lead to IS persistence.

**Results:**

We show through a simulation model that ISs are gradually eliminated from a population even if IS transpositions occasionally cause advantageous mutations. With beneficial mutations, transposition bursts create variation in IS copy numbers and improve cell fitness on average. However, these benefits are not usually sufficient to overcome the negative selection against the elements, and transposition bursts amplify the mean fitness effect which, if negative, simply accelerates the extinction of ISs. If down regulation of transposition occurs, IS extinctions are reduced while ISs still generate variation amongst bacterial genomes.

**Conclusions:**

Transposition bursts do not help ISs persist in a bacterial population in the long run because most burst-induced mutations are deleterious and therefore not favoured by natural selection. However, bursts do create more genetic variation through which occasional advantageous mutations can help organisms adapt. Regulation of IS transposition bursts and stronger positive selection of the elements interact to slow down the burst-induced extinction of ISs.

**Electronic supplementary material:**

The online version of this article (doi:10.1186/s12862-015-0560-5) contains supplementary material, which is available to authorized users.

## Background

Insertion sequences (ISs) are simple, widely observed mobile genetic elements that only contain genes related to transposition and the regulation of transposition [1–5]. Transposition events can shift, replicate, or delete copies of ISs within a genome, which may induce mutations that change fitness of the host cell [3, 6, 7]. IS-induced mutations are often deleterious [5, 7], which raises the question of how those elements became abundant in a bacterial population [8–10]. To answer this question we need to understand how IS dynamics are governed by the rates of IS movement and resulting mutational effects.

Several hypotheses have been proposed to explain the persistence of mobile DNA in a prokaryote population. The selfish DNA hypothesis asserts that ISs are able to persist through their ability to self-replicate while making no fitness contribution to the genome [11, 12]. Analogous to sex in diploid populations, horizontal gene transfer (HGT) has been considered a major determinant of IS spread among prokaryotic species [13]. However, the role of HGT in maintaining ISs within a population is still under some debate [4, 9, 14, 15]. Condit et al. [16] showed that observed rates of HGT are too low to maintain ISs as parasites. Bichsel et al. [17] used a model to point out that although the rate of HGT is generally small, if the fitness cost of an IS is even smaller, HGT allows ISs to invade and persist for long periods in an asexual population with low probability. Bichsel et al. [18] proposed that occasional beneficial IS-induced mutations may be important in order to reach observed IS distributions in a realistic period of time.

An alternative to the selfish DNA hypothesis is that adaptive mutations play a vital role in the persistence of ISs [4, 18, 19]. ISs have been considered as a source of genetic diversity [15, 20], because IS movements mediate changes that are sometimes beneficial to their host genomes [21–23]. If these beneficial mutations prevail in a population through natural selection, ISs can hitchhike to fixation alongside them [24, 25]. Experimental evidence has shown that ISs can increase organismal fitness and thus promote adaptive evolution [6, 26–28]. One important example of this phenomenon is the spread of ISs in bacterial species under antibiotic exposure as the elements are involved in the expression and mobilisation of antibiotic resistance genes, which has been a focus of research in recent decades [29–34]. Using a simulation model Edwards and Brookfield [35] showed that mobile DNA sequences can be maintained in a clonal species if transient beneficial insertions appear for sufficiently long periods of time in one of two alternating environments. Following this work, McGraw and Brookfield [36] derived an optimal transposition rate for element maintenance if reversible advantageous mutations exist in a fluctuating environment.

Mobile elements may also randomly drift to fixation in bacterial genomes [37, 38]. The effect of drift becomes weaker as the size of the population increases [37, 39], and bacterial populations may be large enough to efficiently eliminate mobile elements that cause detrimental effects [40]. However, if the fitness costs induced by IS movements are small or close to neutral, ISs would be hard to eliminate [41]. A study of large-scale genomic data showed that empirical distributions of IS elements are compatible with selective neutrality of elements and a high deletion rate [38]. The three major hypotheses, namely the selfish DNA, the adaptive and the neutral hypotheses, should not be regarded as mutually exclusive for explaining the abundance of ISs, given the complicated interactions among horizontal gene transfer, the distribution of fitness effects, drift, and the rate of transposition [41–43].

Both IS elements and their hosts can encode mechanisms that suppress transposition activities [4, 28, 44–46]. The rate of IS transposition is often suppressed through such negative regulation [3]. The release from regulation of IS transpositions in response to environmental challenges has been observed [33, 45, 47–52]. We call this elevation of the transposition rate a *transposition burst*. An IS transposition burst can promote the adaptation of *Escherichia coli* to a high-osmolarity environment by increasing the rate of beneficial mutations [53]. These changes in transposition activities are sometimes understood as stress responses to changing environments [45, 48, 54] and suggest another hypothesis: periodic transposition bursts increase the IS copy number and thereby promote the persistence of ISs [51]. However, increased transposition rates can also lead to an accumulation of deleterious mutations in the genome. The amplification of fitness costs induced by erratic IS movements may even drive the host population to extinction via the Muller’s ratchet effect [40, 41, 55, 56]. Following the occurrence of transposition bursts, regulation of bursts may evolve and limit transposition rates during an IS invasion [57–61]. Using a simulation model of transposable elements in sexual diploid populations, le Rouzic and Capy [51] suggested that an initial burst followed by strong regulation of transposition can lead to successful invasion of transposable elements.

In this study, we examine whether transposition bursts and the regulation of bursts help to maintain ISs in an asexual population. We investigate how various mutational effects mediated by IS movements interact with transposition rates to influence the dynamics of ISs. Transposition bursts may create more IS copies along with genomic diversity which is essential for bacterial evolution. On the other hand, the extra deleterious IS-induced changes is a net burden for host cells. We show how cells carrying ISs are often eliminated by the population even with the possibility of generating advantageous mutations.

## Methods

We introduce a simulation model of the movement of ISs within genomes and consider the impact of movement on cell fitness and population dynamics. Let the bacterial population be of constant size *N*. Let *x*_*i*_ be the frequency of cells with arrangement *i* where *i*=1,…,*n* and where *n* is the number of different arrangements of IS elements in the population (*n* is a dynamic variable). Each arrangement *i* is associated with a fitness *w*_*i*_ and a copy number *l*_*i*_ of IS elements. The total number of IS copies in a population at time *t* is $\sum _{i=1}^{n} x_{i}(t) l_{i}$.

### Reproduction

In reality, cell generations overlap and there is variation in the time taken for cells to divide. Rather than using a model of binary fission, we let cells reproduce clonally according to the Wright-Fisher process with natural selection and measure time discretely in generations *t*. This assumes that one generation is the average time for cellular reproduction. The vector of IS arrangement frequencies in generation *t*+1 is thus

$$\begin{array}{*{20}l}  \textbf{x}(t + 1) \sim \text{Multinomial} \left(N, \frac{w_{1}(t)x_{1}(t)}{\bar{w}(t)}, \dots, \frac{w_{n}(t)x_{n}(t)}{\bar{w}(t)} \right) \end{array} $$

where $\bar {w}(t) = \sum _{i} w_{i}(t)x_{i} (t)$ is proportion to the mean fitness in the population.

### Transposition events

Within a cell, we define three transposition events that affect ISs. The underlying transposition parameters (*θ*, *μ* and *ν* defined below) are constant per element, per cell, per generation for all cells. But we introduce a factor *λ*(*t*) which scales the underlying transposition probabilities and allows the transposition rates to change over time. With probability *θλ*(*t*) an IS may *shift* to a new location in the cell. With probability *μλ*(*t*) an IS *insertion* event duplicates the IS by adding a new copy at another location in the genome. With probability *νλ*(*t*) an IS *excision* event deletes an IS element from a cell.

The numbers of shift, insertion and excision events in a cell of arrangement *i* are distributed as Poisson with parameters *λ*(*t*)*θl*_*i*_, *λ*(*t*)*μl*_*i*_ and *λ*(*t*)*νl*_*i*_ respectively. For each event a new genome arrangement is created (*n* is set to *n*+1) and the count of the arrangement that experienced the event is decremented by 1. Under a shift event the copy number *l*_*i*_ is preserved in the new *n*^*th*^ arrangement, but the fitness of the new arrangement *w*_*n*_ may be altered as described below. Under an insertion event the copy number increases by one and under a deletion event the copy number decreases by one. Again the fitness of the new arrangement may be altered relative to the original arrangement. We cap the number of ISs per cell by setting a maximum of 100 insertion sites.

### Regulation of bursts

Transposition probabilities may increase when a bacterial population faces stressful environments [26, 49]. We describe this situation as a transposition *burst*, and model the strength of an initial burst using parameter *δ*≥0. We allow *λ*(*t*) to decrease with time after the initial burst at rate *γ*. This decrease models the action of natural selection lowering the deleterious effects of transposition. Thus

(1) $$\begin{array}{*{20}l} \lambda(t) = \begin{cases} 1 \text{, for \(t < T\)} \\ 1 + \delta e^{- \gamma (t - T)} \text{, for \(t \geq T\)}  \\ \end{cases} \end{array} $$

where *T* is the time when changes in transposition activities occur.

**Fig. 1:**
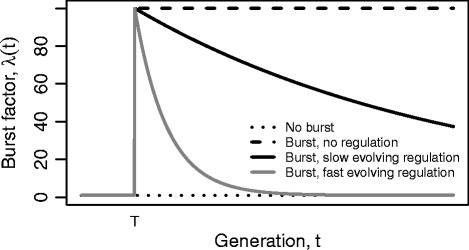
Three scenarios for scaling the global transposition probabilities. The burst factor *λ*(*t*) is given by Eq. (1). *No burst* (dotted): the transposition probabilities (*θ*, *μ* and *ν*) are constant for all cells, *δ*=0, *γ*=0. *Burst, no regulation* (dashed): there is a continuous burst in transposition activities, occurring at time *t*=*T*, that scales the basic probabilities by 100, *γ*=0. *Burst, evolving regulation* (solid): cells evolve regulation in transposition activities after the burst has occurred, the transposition probabilities asymptotically approach the global transposition probabilities over time, *γ*=0.001 (*black solid*) and 0.01 (*grey*). Other parameter values are as given in Table 1

Figure 1 illustrates three possible scenarios that we consider for the change in transposition probabilities. Under the first scenario (*no burst*), there is no burst of transposition (*δ*=0) so that *λ*=1 for all *t* and all cells in the population always have the same underlying transposition probabilities (dotted). Under the second scenario (*burst, no regulation*), a transposition burst event occurs (*δ*>0) at time *t*=*T* and transposition rates are permanently elevated from that time (dashed). In the third scenario (*burst, evolving regulation*) a transposition burst occurs at time *t*=*T* which is followed by the gradual evolution of down-regulation of transposition rates so that the original underlying transposition probability is approached asymptotically (solid). The speed of this process of evolved intracellular down-regulation is controlled by parameter *γ* which is large for fast evolution (grey) and small for slow evolution (black solid).

**Table 1:** Parameters with default values

Symbol	Description	Default value
*N*	Population size	10^5^
*θ*	Shift probability per IS per cell per generation	10^−6^
*μ*	Insertion probability per IS per cell per generation	10^−6^
*ν*	Excision probability per IS per cell per generation	10^−6^
*δ*	Initial burst strength	99
*γ*	Evolutionary rate of regulator	10^−3^
*a*	Magnitude of fitness benefit per transposition event per cell generation	2×10^−4^
*d*	Magnitude of fitness cost per transposition event per cell generation	2×10^−4^
*p*_*a*_	Probability of IS fitness benefit	0.05
*p*_*d*_	Probability of IS fitness cost	0.8

### Fitness effects

A transposition event induces a fitness change whose effect on the new cell may be positive, neutral, or negative. An event produces an advantageous change with probability *p*_*a*_ and a deleterious change with probability *p*_*d*_. The magnitudes of the selective effects are *a* for advantageous changes and −*d* for deleterious changes. The IS copy number of a genome of type *i* can be partitioned into numbers of elements that produced advantageous (*l*_*i*,*p*_), neutral (*l*_*i*,*n*_) and deleterious (*l*_*i*,*d*_) effects, so that *l*_*i*_=*l*_*i*,*a*_+*l*_*i*,*n*_+*l*_*i*,*d*_. In our model for IS-induced fitness changes, we assume that excision or shift events reverse the fitness effect that the IS originally induced. If a transposition event occurs in cell type *i* the new arrangement is given a new index *n*^′^ and has fitness given by:

(2) $$\begin{array}{*{20}l} n' &= n+1  \\ w_{n'} &= (1+a)^{l_{a}} (1-d)^{l_{d}} \end{array} $$

where the prime (^′^) indicates the new value of the variable *n* after the new arrangement is generated (after this operation *n* is set to *n*^′^).

### Initialisation

Several studies have analysed the distribution of IS copy number in bacterial genomes and observed similar count distributions [9, 14, 18]; the published distributions of IS copy number are right skewed with some distributions highly right skewed. Sawyer et al. [62] reported distributions of six unrelated ISs in a collection of 71 *Escherichia coli* strains from various natural isolates. For our numerical work we use the distribution of *IS*5 from Sawyer’s study, namely, ***α***=(0,1,2,3,4,5,6,9,21) IS copies in ***B*** =(46,12,3,2,2,2,2,1,1) genomes. This reflects a typical IS copy number distribution in bacterial populations, where a majority of the genomes contain no ISs and a moderate number of cells contain higher numbers of ISs. We define ***β*** to be the distribution of IS copy number in a population and initialise the distribution of ISs in the population with ***α*** ISs in ***β*** cells, where ***β***∼Multinomial(*N*,**B**/71). We also consider a second scenario where the population is initialised with a single IS element in a single cell, so that we can investigate the impact of transposition bursts on the initial invasion of IS elements.

### Simulation and parameters

At each generation, cell reproduction is followed by potential IS transposition events. For any cell type *i* where *i*=1,…,*n*, these processes may lead to new cell types with new fitness based on current fitness *w*_*i*_, and with a new IS copy number based on *l*_*i*_. Unless otherwise specified, we use the parameter values shown in Table 1. Observed rates of IS transposition are approximately *θ*, *μ*, *ν*=10^−8^ per cell generation [3, 63]. To expedite computation while preserving the same rate of supply of transpositions [39], we set a default population of size 10^5^ with transposition rates *θ*, *μ*, *ν*=10^−6^.

We set the magnitude of positive and negative selection coefficients per IS per cell per generation, *a* and *d*, to range broadly from 10^−5^– 10^−3^ because empirical estimates of these parameters are scarce and cover a wide range [7, 17, 18]. We assume that an IS-induced mutation is beneficial to its host cell with probability *p*_*a*_=0.05, deleterious with probability *p*_*d*_=0.8, and neutral otherwise; this is based on estimates of effects of insertional mutations in Elena et al. [7]. By varying the value of *p*_*a*_ and adjusting the other probabilities correspondingly at the same time that the transposition burst occurs, we also simulate a scenario where a new environmental condition changes the distribution of IS-mediated mutational effects.

### Output variables

In each simulation run, we record the following output variables in the population over time: the proportion of cells carrying ISs with the proportion of IS copies in advantageous, deleterious and neutral sites; the mean IS copy number per cell; and the mean fitness of cell population. We track the 5 *%* and 95 *%* quantiles of IS copy numbers carried by a cell in the population, which indicate the range of IS counts in genomes. We also track the 5 *%* and 95 *%* quantiles of cell fitness in the population to examine the impact of IS transpositions on the organismal fitness. From 1000 simulation runs, we calculate the probability of IS extinction by computing the proportion of runs in which all ISs are eliminated by the population. At the end of 30,000 simulated generations, we describe the distribution of ISs in the population by calculating the mean proportion of cells carrying ISs, mean IS copy number per cell and the mean fitness of cell population across multiple runs. We increase the number of simulation runs to 10,000 when investigating the invasion of a single IS element in a population with no ISs.

## Results

We study IS extinction and persistence over 30,000 generations under three settings of transposition rates (shown in Fig. 1). In the first scenario (*no burst*), transposition occurs at a relatively low and constant rate. In the second scenario (*burst, no regulation*), we introduce a 100-fold step change in transposition probability at the 5000^*th*^ generation. We use this scenario to model a burst in transposition activities which may be induced by a sudden change in the environment. In the third scenario (*burst, evolving regulation*), we introduce a transposition burst at the 5000^*th*^ generation but also allow regulation of the transposition burst to evolve over time.

**Fig. 2:**
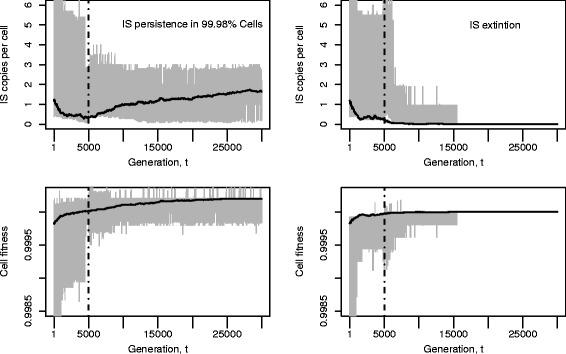
Two simulation runs showing changes in IS copy number and cell fitness under transposition burst with evolving regulation. Transposition burst occurs at 5000^*th*^ generation, and is gradually down-regulated over time thereafter. ISs may persist (*left*) or go extinct (*right*) at the end of 30,000. *Top* panels: mean IS copy per genome in the population is drawn in black with its 5 *%* and 95 *%* quantiles coloured in *grey*. *Bottom* panels: mean fitness of the population increases over time (*black*) with its 5 *%* and 95 *%* quantiles coloured in grey. The population is initialised with *α* ISs in *β* cells according to Sawyer et al. [62]. Other parameter values are as given in Table 1

Figure 2 illustrates the dynamics under a transposition burst with evolving regulation. Two realisations of the simulation over 30,000 generations under the same parameter settings show that ISs may persist in the population (left) or go extinct (right). The grey region in the two top panels shows the central 90 *%* range of IS copies and an increase in variation can be seen after the burst in transposition rate at the 5000^*th*^ generation. The mean fitness of the population gradually increases over time in both cases of persistence and extinction (black curve, bottom panels).

**Fig. 3:**
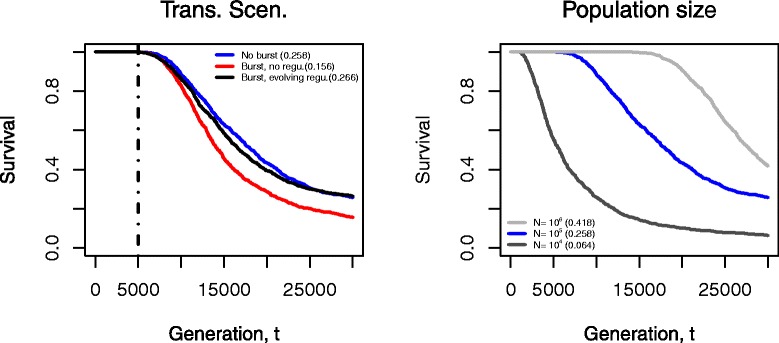
Survival curves under different scenarios of transposition activities and population sizes. All changes in transposition events occur after 5000 generations (vertical lines). In the left panel, we compare the three settings in transposition rates. The *right* panel demonstrates the influence of different population sizes. Transposition bursts lead to more IS extinctions (*red*), and down-regulation of the bursts can lead to IS survival that is at a similar level to *no burst* scenario (*black and blue*). Each curve was calculated based on 1000 simulation runs, and each simulation was run for 30,000 generations. The survival probability of ISs for each curve at the end of simulations was given in parentheses. Parameters: *No burst* (*blue*): *δ*=0, *γ*=0. *Burst, no regulation* (*red*): *γ*=0. Population size *N*=10^4^ (*dark grey*) and 10^6^ (*light grey*). The population is initialised with an IS distribution according to Sawyer et al. [62]. Other parameter values are as given in Table 1

We calculated the survival curve of ISs in a population from 1000 runs under different settings of transposition rates and population sizes (Fig. 3). The step change in transposition rates is shown as a vertical line at the 5000^*th*^ generation. The left panel shows that constant and relatively low transposition rates (*no burst*) lead to roughly 26 *%* IS survival by the end of the simulation under the default settings (blue). If bursts are unregulated the probability of survival of ISs is reduced to around 16 *%* (red). When transposition bursts are gradually down-regulated (black), it is possible for the survival probability of ISs to reach a similar level as in the *no burst* scenario. Although only reversible IS-induced effects have been considered in this model, it is also possible for IS movements to “leave behind” the original fitness effects. Therefore, in the Additional file 1 (Section 1), we provide comparison between reversible and irreversible changes for their impact on the survival of ISs. Although the reversibility of mutational effects improves the survival of ISs, the effect appears to be slight. In contrast, the size of the population has a strong influence on the survival curve as shown in the right panel of Fig. 3.

**Fig. 4:**
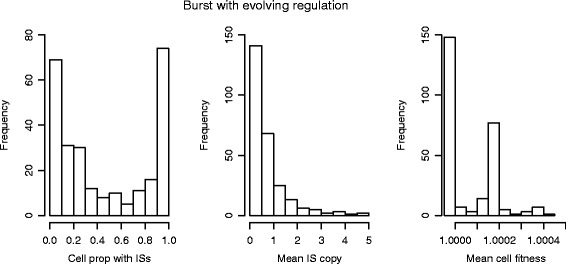
Final distribution of ISs and cell fitness under transposition bursts with evolving regulation. All changes in transposition events occur after 5000 generations. Each panel was generated based on 1000 simulation runs, and each simulation was run for 30,000 generations. The mean IS copy and the mean cell fitness were computed conditional on the persistence of ISs. The population is initialised with an IS distribution according to Sawyer et al. [62]. All parameter values are as given in Table 1

We considered features of populations in which ISs survived to 30,000 generations in the case of transposition bursts with evolving down-regulation. Figure 4 shows the distribution of the proportion of cells carrying any IS elements, the mean IS copy number and the mean fitness across 1000 simulations. In about 28 *%* of the simulation runs the proportion of cells carrying ISs was greater than 90 *%* while the distribution formed from the remaining simulations was right skewed (left). The distribution of the mean IS copy number is right skewed (middle). The cell fitness distribution is also right skewed and mostly above 1 (right) due to the selection of beneficial mutations. All corresponding results under other transposition scenarios and reversible mutational effects are provided in the Additional file 1 (Section 2). From this point onwards, we focus on the model of transposition bursts with evolving down-regulation unless otherwise specified.

### Role of transposition bursts and their regulation

**Fig. 5:**
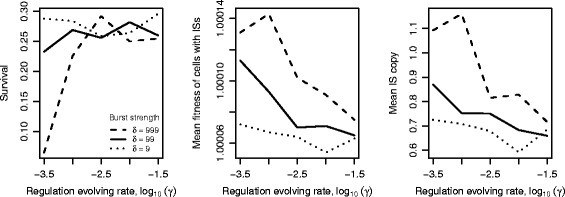
The roles of burst strength *δ* and rate of regulation evolution *γ* on IS persistence. All changes in transposition events occur at the 5000^*th*^ generation. Each pair of *δ* and *γ* was simulated for 1000 runs, and each simulation was run for 30,000 generations. The mean IS copy and the mean cell fitness were computed conditional on the persistence of ISs. Parameters: *δ*=9,99,999, and *γ*∈ [ 10^−3.5^,10^−1.5^]. The population is initialised with an IS distribution according to Sawyer et al. [62]. All parameter values are as given in Table 1

Figure 5 shows the effects of the strength of burst *δ* and the rate of evolution of burst regulation *γ* on the survival of ISs (left), the mean cell fitness (middle) and the mean IS copy number per cell (right) after 30,000 generations. As the rate of evolution of regulation increases, ISs are more likely to persist but with lower mean cell fitness and mean IS copy per cell. This pattern is most evident in the case of strong bursts (*δ*=999), where down-regulation of transposition is expected to have the greatest effect. We highlight the observation that strong bursts with weak regulation (*δ*=999, *γ*=10^−3.5^) produce not only low survival of ISs but also high mean fitness and high IS copy numbers. This phenomenon is due to a selection bias whereby cells that survive the burst tend to be the ones with high fitness. The burst itself elevates copy number.

### Role of fitness effect distribution

**Fig. 6:**
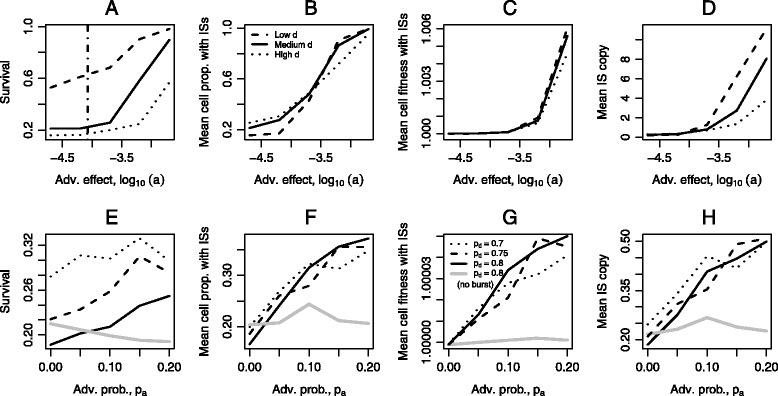
The role of different fitness distributions on IS persistence. The mean cell proportion with ISs, the mean cell fitness and the mean IS copy were computed conditional on the persistence of ISs. *Top*: Transposition bursts with evolving regulation occur after 5000 generations; the magnitude of positive (*a*) and negative (*d*) selective forces are given in the horizontal axis and legends respectively. The persistence of ISs is significantly affected by the magnitude of beneficial selection, and an estimated threshold value of *a*=10^−4.07^ (vertical dashed line) calculated based on Eq. 3 reasonably predicts when ISs would be lost. *Bottom*: There is no beneficial IS-induced mutation (*p*_*a*_=0) during the first 5000 generations; transposition bursts with evolving regulation and increase in *p*_*a*_ occur thereafter except for the blue curve (*no burst*, *δ*=*γ*=0), corresponding values of *p*_*d*_ for each curve are provided in the legends. IS persistence is favoured by transposition bursts and more IS-induced adaptive mutations. Each pair of parameters was simulated for 1000 runs, and each simulation was run for 30,000 generations. The population is initialised with an IS distribution according to Sawyer et al. [62]. Other parameter values are as given in Table 1

In Fig. 6, we investigated the effects of the magnitudes of positive *a* and negative *d* selection (top), and the effects of the probabilities of advantageous *p*_*a*_ and deleterious *p*_*d*_ mutations (bottom) on the distribution of ISs and the mean cell fitness at the end of simulations where the ISs did not go extinct. As expected, low deleterious effects *d* and high advantageous effects *a* generally lead to greater survival of ISs and greater mean fitness. The threshold value of *a* below which IS are expected to be lost is presented as a vertical dashed line in panel A, and the derivation of this threshold is provided in the end of ‘Results’ section (Eq. 3). While *a* also influences the mean proportion (panel B) and the mean fitness (panel C) of cells carrying ISs, the deleterious effect size *d* does not.

We varied *p*_*a*_ and *p*_*d*_ to examine the case where a change in the environment makes available new beneficial mutations to be generated by IS transpositions (Fig. 6, bottom panels, black). In terms of the model, the value of *p*_*a*_ changes from 0 to positive values after 5000 generations. We find that the mean cell proportion carrying ISs is gradually improved as *p*_*a*_ increases (panel F, black), as are the mean population fitness (panel G, black) and the mean IS copy per cell (panel H, black). For comparison we also examined whether IS persistence is still favoured by more IS-induced adaptive mutations in the absence of bursts in transposition rates (Fig. 6, bottom panels, thick grey). We find that increase in the probability of fitness benefit alone does not help ISs to persist in a population.

### Invasion by a single IS element

**Fig. 7:**
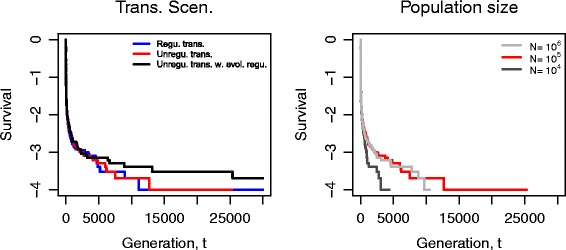
Survival curves for invasions by a single IS element under different scenarios. Left and middle: Unregulated transposition activity (*red*) and that with evolving regulation (*black*) both occur at the same time as the IS element enters the population. We compare them with the case of constant transposition rates (*blue*). Unregulated transposition activities, regardless of further regulation and mechanism of mutations (non-reversible *vs.* reversible), do not affect the survival of a single IS element. Right: The strength of selection does not influence the survival of ISs. Each curve was calculated based on 10,000 simulation runs, and each simulation was run for 30,000 generations. Blue and grey: *δ*=0, *γ*=0, *N*=10^4^ (*dark grey*) and 10^6^ (*light grey*). *Red*: *γ*=0. Other parameter values are as given in Table 1

Theoretical studies propose that a lack of regulation in transposition activities may contribute to a successful invasion of ISs in a sexual diploid population [51]. To examine this idea for asexual organisms, we introduce one IS element in a genome of a bacterial population. Simulation results indicate that the element usually goes extinct quickly (Fig. 7), in agreement with the analyses of Bichsel et al. [17]. According to our model, unregulated transposition does not affect the survival of a single IS element, regardless of whether down-regulation evolves (red and black), whether mutations are reversible or not (left and middle), or whether the strength of selection is increased by increasing the population size (right).

### Heuristic model of persistence of IS elements

To explain the broad patterns of IS persistence, we consider here a simplified version of the simulation model to find conditions under which IS elements are expected to persist evolutionarily. Let us ignore polymorphism and assume deleterious mutations do not reach fixation. Let *p*_*n*_ be the proportion of IS-induced changes that are selectively neutral.

Under the simulation model, the rate of supply in the population of advantageous changes that lead to an increase in copy number is *λ*(*t*)*μp*_*a*_*N*. The supply rate of neutral changes leading to an increase in copy number is *λ*(*t*)*μp*_*n*_*N*. The supply rate of changes leading to a decrease in copy number is *λ*(*t*)*νp*_*n*_ assuming that the loss of elements does not change fitness. The probability of fixation is approximately 2*a* for advantageous changes [64] and 1/*N* for neutral changes [65]. (Here consider only one change at a time and ignore deleterious insertions in the background of advantageous insertions). Therefore the rate of substitution of copy-increasing events is

$$ K_{(+)} = 2 a \lambda(t)\mu p_{a} N + \lambda(t) \mu p_{n} $$

and the substitution rate of copy-decreasing events is

$$ K_{(-)} = \lambda(t) \nu p_{n}. $$

Thus, while a burst elevates the rate of increase of IS copies, it also elevates the rate of decrease. Since zero copies is an absorbing boundary, increasing transposition via *λ*(*t*) speeds up the extinction of ISs through increased *K*_(−)_. However, the rush to extinction due to a burst is mitigated by the evolution of down-regulation. Assuming ISs do not go extinct near this zero boundary a rough criterion for the long term success of ISs is given by the ratio *K*_(+)_/*K*_(−)_ being greater than unity. That is, IS persistence requires

(3) $$\begin{array}{*{20}l} \frac{\mu (2 a p_{a} N + p_{n})}{\nu} &> 1.  \end{array} $$

Because the burst function *λ*(*t*) is cancelled out in the above threshold it ultimately does not influence the long term success of ISs except in hastening their initial extinction when near the zero boundary.

The condition (3) is similar in form to the persistence condition for mobile elements given by Lynch [66]. The differences are that we disregard deleterious changes (which are also eventually neglected in Lynch’s treatment), and we include a term for adaptive changes. Adaptive changes play a role when the product *ap*_*a*_ is large enough when compared to the reciprocal of the population size, 1/*N*.

In summary, the success of insertion sequences depends not only on the balance between new insertions and deletions but also on the strength of positive selection of IS-induced changes which in turn depends on both population size and the distribution of fitness effects, as revealed in expression (3).

## Discussion

In this study we have investigated the role of transposition bursts on the survival of ISs in a bacterial population by modelling the effects of IS movements on the fitness of host genomes. Bursts in transposition activity hasten the extinction of mobile elements because they increase both excision events and IS-induced fitness costs. In other words, elevated transposition serves to amplify both deleterious and advantageous fitness changes but this accelerates the extinction of elements, particularly if the mean fitness effect of IS-induced mutations is negative. However, transposition bursts create genetic diversity which occasionally generates advantageous mutations and thus help organisms adapt to new environments. If transposition activity is eventually down-regulated following a transposition burst, the elimination of ISs from a population is slowed down.

Because transposition bursts can rapidly increase the number of IS copies, it has been proposed that bursts favour the invasion of ISs [51, 67]. We find, however, that since invading ISs are initially rare and close to the extinction boundary, they are already vulnerable to quick extinction [17]. The invasion of ISs in an asexual population is not helped by bursts.

Transposition bursts have been postulated as a stress response for bacterial populations facing environmental challenges [23, 49, 68]. Bursts in IS transposition have been reported to be associated with harsh environmental conditions [45, 48, 50]. As an example, antibiotic treatment results in an increased frequency of IS transposition which may accelerate the development of resistance [33, 52]. Under our model, strong transposition bursts increase the mean fitness of organisms through selection of beneficial mutations. Bacteria can occasionally benefit from such increased genetic variability which allows for adaptation to new environments [49, 69]. However, the amplification of deleterious mutations increases the risk of host lineage extinction [40, 55]. It also favours genomes lacking IS elements. If a population eliminates ISs through natural selection, it loses the ability to use IS elements for adaptation under environmental stress in the future. Hence, transposition bursts can accidentally promote bacterial evolution but they are unlikely to have evolved as an adaptive strategy for either ISs or genomes. Transposition bursts alone cannot explain the abundance of ISs over long-term periods of time.

Regulation of transposition bursts decelerates the burst-induced extinction of ISs while creating some genomic variability. Both IS elements and hosts appear to have evolved regulatory mechanisms that limit transposition activities [44, 49, 59]. Natural selection favours bacterial hosts that evolve the ability to suppress transposition bursts in response to the amplified effects of deleterious mutations [57, 68]. Transposition bursts with evolving regulation represent a compromise between providing a host population with a means of adapting to new environments on one hand and promoting the persistence of ISs on the other [51]. However, if the mean fitness effect on their hosts is negative, a given family of IS elements may still go extinct eventually.

Advantageous effects mediated by IS transposition may serve as a necessary condition for the spread of ISs in prokaryotes [4, 18]. ISs can be viewed as mutator genes whose prevalence increases in bacterial populations over successive selective sweeps as they hitchhike with beneficial mutations that they produce [35, 70], as confirmed in experimental studies [24, 26]. Our model shows how benefits of occasional adaptive mutations can be amplified by transposition bursts and overcome intermittent drops in the mean population fitness. With an initial IS distribution with a low mean copy number [62], we find that ISs gradually go extinct in the population, and the process of IS elimination is slower if positive selection on beneficial mutations is strong or if the transposition rate is low. A high mean fitness effect of IS movements can be achieved by either IS-induced mutations carrying higher fitness benefits or higher rates of beneficial mutations. How those mutational effects vary and change the distribution of ISs in genomes under environmental stress is a topic worth investigating experimentally. Previous studies have shown that reversible IS-induced mutations with precise excisions may benefit cells in fluctuating environments [35, 36]. We have compared non-reversible versus reversible mutation effects for their influence on the persistence of ISs; although reversibility may be beneficial the difference between the two models is slight.

Our model has been kept simple to focus on the effect of transposition bursts on the dynamics of ISs. However, it could be made more biologically realistic by including recombination. Given that ISs can promote recombination in bacterial strains [71], one can include recombination in future models to study how it influences the IS dynamics by creating more genomic variation. Recombination may also cause deletions of ISs [71]. Genomic evidence has shown that transposable elements may go extinct periodically in bacterial lineages [14]. A single IS element that is newly introduced in a population with no other ISs can rapidly go extinct due to drift and purifying selection [17]. Therefore horizontal transfer of ISs from other species is essential for the initial introduction and re-introduction of IS elements [10, 13, 17, 40, 66]. Alternatively, ISs can be maintained in a population if the intraspecific horizontal transfer rate is high. Over evolutionary time scales, ISs can only be maintained in a bacterial population if the acquisition and establishment of mobile elements can outrun their elimination. The rate of IS elimination in turn can be decreased through advantageous mutations and the regulation of transposition bursts. It would be interesting to further study the balance of these processes.

## Conclusions

We find that transposition bursts do not help insertion sequences persist; rather, they accelerate the elimination of ISs from populations because IS-induced changes are mostly harmful to host genomes. Since ISs are often lost during transposition bursts, we do not consider ISs or transpotion bursts to have evolved as an adaptive strategy to deal with environmental stress. However, bacteria in new environments can occasionally benefit from the genetic variation generated by bursts in IS transposition activity, and the burst-induced extinction of ISs can be decelerated by evolved down-regulation of transposition.

## Availability of supporting data

We provide the computer simulation code used in our study in the Additional file 1, Section 3.

## Additional file

Additional file 1
**The additional file contains the following sections.** Section 1 describes the impact of mutational reversibility on the persistence of IS elements. Section 2 compares alternative scenarios for transposition burst and mutation mechanisms. There we provide simulation results showing the persistence of ISs under different conditions. (PDF 344 Kb)
