# Supplementary material for: Dynamics of bacterial insertion sequences: can transposition bursts help the elements persist?
Source: BMC Evol Biol. 2015 Dec 21;15:288. doi: 10.1186/s12862-015-0560-5 (PMC4687120; doi:10.1186/s12862-015-0560-5)
Supplement: Additional file 1 — The additional file contains the following sections. Section 1 describes the impact of mutational reversibility on the persistence of IS elements. Section 2 compares alternative scenarios for transposition burst and mutation mechanisms. There we provide simulation results showing the persistence of ISs under different conditions. (PDF 344 Kb) [file 12862_2015_560_MOESM1_ESM.pdf]

Additional information for *Dynamics of bacterial insertion sequences: can transposition bursts help the elements persist?*

Yue Wu, R. Z. Aandahl and Mark M. Tanaka
School of Biotechnology & Biomolecular Sciences, University of New South Wales

Section 1: The impact of mutational reversibility on the survival of ISs

Here we provide the comparison between different mutational mechanisms for their impact on the survival of ISs. In the irreversible model, excision or shift events do not reverse the fitness change that the IS originally induced. If a shift or insertion event occurs in cell type i the new arrangement is given a new index n' and has fitness given by

$$w_{n'} = \begin{cases} w_i(1+a) & \text{with probability } p_a, \\ w_i(1-d) & \text{with probability } p_d, \\ w_i & \text{with probability } 1-p_a-p_d \end{cases} \quad (1)$$

where

$$n' = n+1$$

where the prime ($'$) indicates the new value of the variable n after the new arrangement is generated (after this operation n is set to n').

The left panel in Figure 1 presents survival when IS-induced mutations are reversible, while the right panel shows survival under irreversible mutational effects. Although the reversibility of mutational effects improves the survival of ISs, the effect appears to be slight.

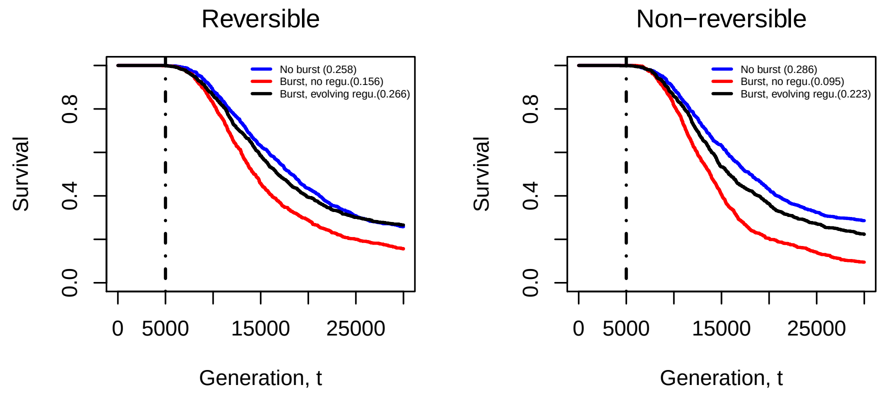

Figure 1 Survival curves under different scenarios of transposition activities and mutational mechanisms. All changes in transposition events occur after $5,000$ generations (vertical lines). We compare the three settings in transposition rates under scenarios of reversible (left) and irreversible (right) mutational effects. Each curve was calculated based on $1,000$ simulation runs, and each simulation was run for $30,000$ generations. The survival probability of ISs for each curve at the end of simulations is given in parentheses. Parameters: *No burst* (blue): $\delta = 0$, $\gamma = 0$. *Burst, no regulation* (red): $\gamma = 0$. The population is initialised with an IS distribution according to Sawyer *et al.* 1987. Other parameter values are as given in Table 1 in the main text.

Section 2: Comparing different transposition scenarios and mutational mechanisms for their impacts on IS persistence

Here we provide results on simulation of IS persistence under different transposition scenarios and mutational mechanisms. Figure 2 demonstrates how the proportion of cells carrying ISs distributes in $1,000$ simulation runs if they did not go extinct at the end of $30,000$ generations. Under the scenario of *no burst* and *burst with evolving regulation*, in a considerable number of runs more than 90% of cells carry ISs, while the distribution of cell proportions carrying ISs is right skewed in the rest of the simulation runs.

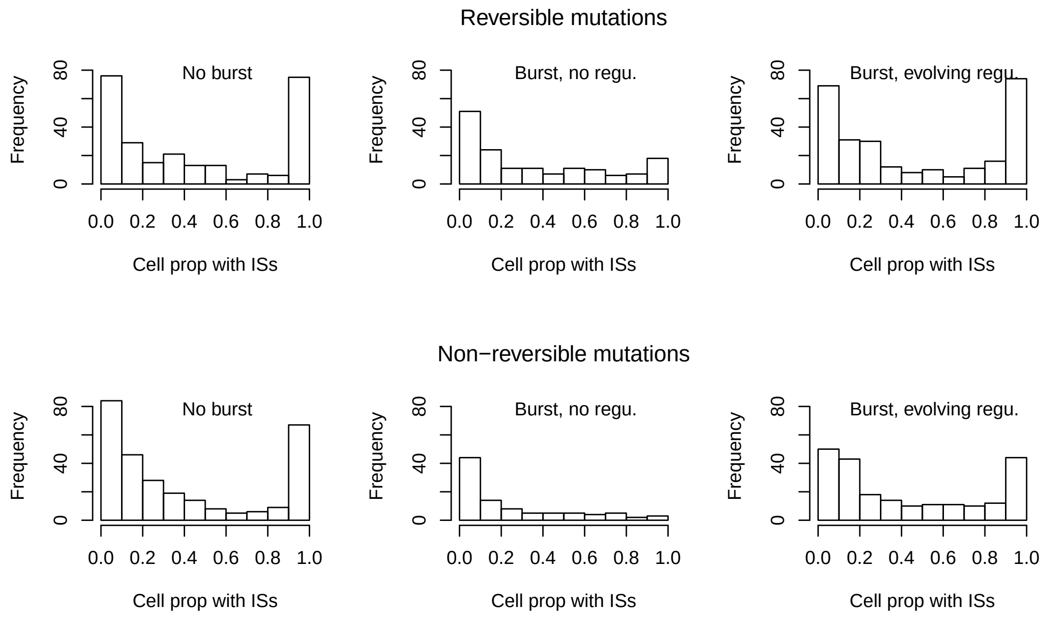

Figure 2 Final distribution of cell proportion carrying ISs under transposition bursts with evolving regulation and non-reversible mutational effects. The mean cell proportion was computed conditional on the persistence of ISs. All changes in transposition events occur at the $5,000^{th}$ generation. Each panel was generated from $1,000$ simulation runs, and each simulation was run for $30,000$ generations. Parameters: *No burst* (left panels): $\delta = 0$, $\gamma = 0$. *Burst, no regulation* (middle panels): $\gamma = 0$. The population is initialised with an IS distribution according to Sawyer *et al.* 1987. Other parameter values are as given in Table 1 in the main text.

Figure 3 and Figure 4 present the distributions of mean IS copy number per cell and the mean cell fitness in the population respectively. Both distributions are right-skewed and not greatly influenced by different transposition activities. There is no apparent difference between non-reversible and reversible mutational effects for their impacts on the persistence of ISs.

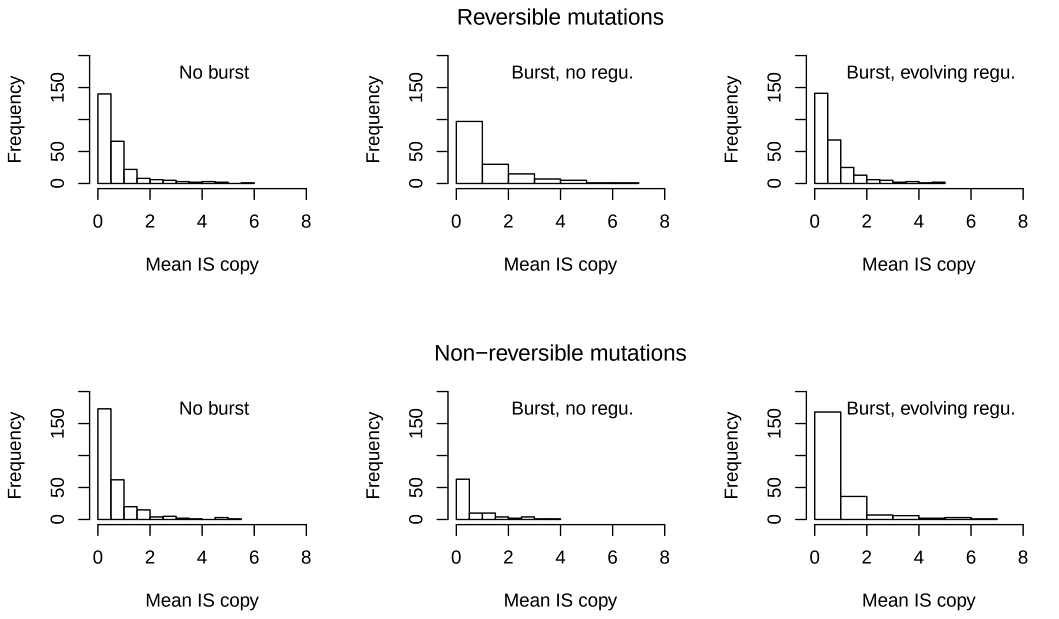

Figure 3 Final distribution of IS copy number per cell under transposition bursts with evolving regulation and non-reversible mutational effects. The mean IS copy was computed conditional on the persistence of ISs. All changes in transposition events occur at the $5,000^{th}$ generation. Each panel was generated from $1,000$ simulation runs, and each simulation was run for $30,000$ generations. Parameters: *No burst* (left panels): $\delta = 0$, $\gamma = 0$. *Burst, no regulation* (middle panels): $\gamma = 0$. The population is initialised with an IS distribution according to Sawyer *et al.* 1987. Other parameter values are as given in Table 1 in the main text.

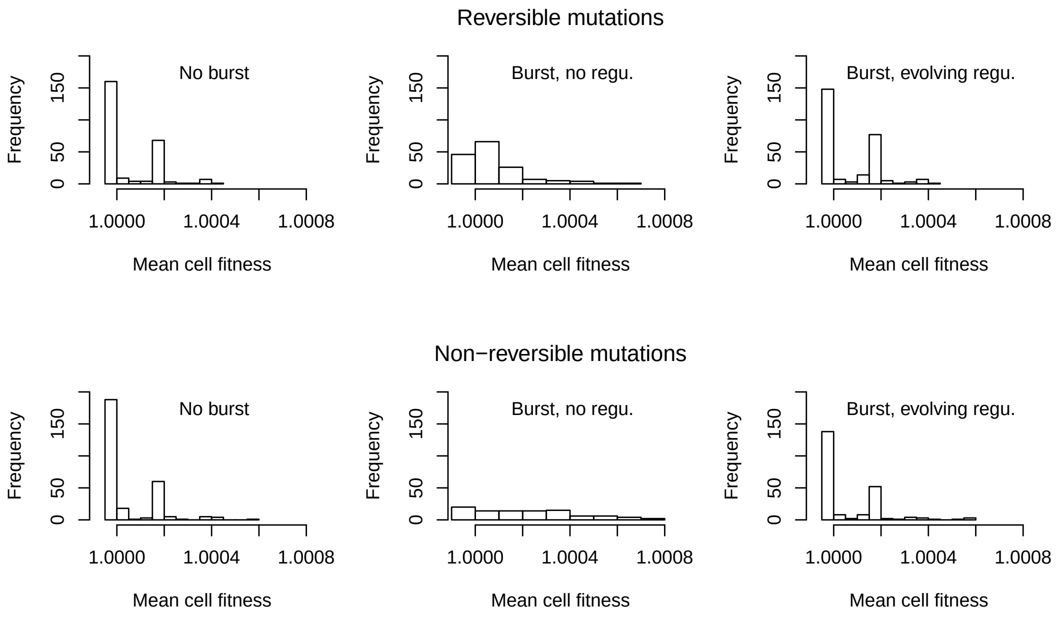

Figure 4 Final distribution of cell fitness under transposition bursts with evolving regulation and non-reversible mutational effects. The mean cell fitness was computed conditional on the persistence of ISs. All changes in transposition events occur at the $5,000^{th}$ generation. Each panel was generated from $1,000$ simulation runs, and each simulation was run for $30,000$ generations. Parameters: *No burst* (left panels): $\delta = 0$, $\gamma = 0$. *Burst, no regulation* (middle panels): $\gamma = 0$. The population is initialised with an IS distribution according to Sawyer *et al.* 1987. Other parameter values are as given in Table 1 in the main text.

Section 3: Simulation code

The computer simulation was implemented in R. The R functions appear in the following files whose contents are also provided.

1. SIM-examples.R

```
source("cellMatrixV2.R")

defaultparam <- function() {
sp <- 10^-8 # shift prob
ip <- 10^-7 # insert prob
ep <- 10^-8 # excise prob
sr <- 1
bs <- 0 # burst strength
br <- 0
ap <- 0.05 # adv prob
dp <- 0.8 # del prob
np <- 1-ap-dp # neu prob
af <- 0.01
df <- -0.001
param <- list(sp=sp,ip=ip,ep=ep,
sr=sr,bs=bs,br=br,
ap=ap,dp=dp,np=np,
af=af,df=df)
return(param)
}


# This simulation has a burst at 5000, uses fitness model b with reversible effects
# it returns the time of extinction or the establishment of IS if extinction didn't happen
# The multirun below will run this simulation repeatedly and create a vector of the output
runEx1 <- function(x) {
param <- defaultparam()
paramb <- param
paramb$bs <- 99 # burst changes
paramb$br <- 10^-3 # burst changes
fitModel = "b" # fitness model, b=reversible
testCM <- initialization(N=10^7,param=param)
extinct <- FALSE
t <- 1
while(t <= 20000 && extinct == FALSE) {
if(t==5000) param <- paramb # burst happens at 5000
testCM <- reproduction(testCM)
testCM <- transposition(testCM,param,fitModel=fitModel)
testCM <- compressCellMatrix(testCM)
testCM <- updateRegulator(testCM,param)
if(extinction(testCM)) extinct <- TRUE
t <- t+1
}
if(extinct) return(t) # returns an integer with time if extinction happens
return(establishment(testCM)) # if time runs out: a real between 0 and 1 for establishment
}

# this returns a vector of output for 10 runs from runEx1
multiRunEx1 <- function() {
out<-do.call(c,lapply(seq(1,10),runEx1))
return(out)
}

# how to use
# out <- multiRunEx1()
# plot(out[out<1])          this plots the simulations that didn't go extinct (establishment ratio)
# plot(out[out>1])          this plots simulations that went extinct (extinction times)
# sum(out<1)/length(out)    the percentage of extinctions
# sum(out>1)/length(out)    the precentage of non-extinctions



# This simulation has a burst at 2000, uses fitness model a without reversible effects
```

```
# it tracks, min/max/mean IS and min/max/mean fitness, proportion IS (adv,del,neu) over time (matrix output)
runEx2 <- function(x) {
param <- defaultparam()
paramb <- param
paramb$bs <- 99 # burst changes
paramb$br <- 10^-3 # burst changes
fitModel = "a" # fitness model, b=reversible
testCM <- initialization(N=10^7,param=param)
extinct <- FALSE
t <- 1
outVec <- NULL
while(t <= 5000 && extinct == FALSE) {
outVec <- rbind(outVec,c(getMinIS(testCM),getMaxIS(testCM),getMeanIS(testCM),getMinFit(testCM),
getMaxFit(testCM),getMeanFit(testCM),getPropIS(testCM),establishment(testCM)))
if(t==2000) param <- paramb # burst happens at 5000
testCM <- reproduction(testCM)
testCM <- transposition(testCM,param,fitModel=fitModel)
testCM <- compressCellMatrix(testCM)
testCM <- updateRegulator(testCM,param)
if(extinction(testCM)) extinct <- TRUE
t <- t+1
}
return(outVec) # returns output matrix (minIS,maxIS,meanIS,minFit,maxFit,meanFit,propAdv,propDel,propNeu)
}


# how to use
# out <- runEx2()
# plot(out[,6],type="l")       plots mean cell fitness over time
```

2. cellMatrixV2.R

```
# v2.00
# cellMatrix is Cx5 matrix with (count, adv IS, dis IS, neu IS, fitness, regulator timer)

# initialization sets extant IS to neutral fitness.
# to approximate a small number of founding types with a single IS use initFreq=c(10^7-x,x) where x is something like 15 or so

library(plyr)

source("IS-helper.R")
source("IS-init.R")
source("IS-trans.R")
source("IS-repro.R")
```

3. IS-init.R

```
# v1.00

initialization <- function(N=10^7,param,
                           initFreq=c(46/71, 12/71, 3/71, 2/71, 2/71, 2/71, 2/71,
                             0, 0, 1/71, 0, 0, 0, 0, 0, 0, 0, 0, 0, 0, 1/71),
                           singleFounder=FALSE) {
initDist <- rmultinom(1,N,initFreq)
if(singleFounder) initDist <- matrix(c(N-1,1),nrow=2)
numTypes <- sum(initDist>0)
initCount <- initDist[initDist>0,]
initIS <- which(initDist>0)-1
initISa <- t(do.call(cbind,lapply(initIS,rmultinom,n=1,prob=c(param$ap,param$dp,param$np)))) # according to IS prob
# initISa <- t(do.call(cbind,lapply(initIS,rmultinom,n=1,prob=c(0,0,1)))) # uncomment to set all neutral
fitVals <- matrix(rep(c(1+param$af,1+param$df,1),numTypes),nrow=numTypes,byrow=TRUE)
fitness <- fitVals^initISa
fitness <- apply(fitness,1,prod)
cellMatrix <- matrix(0,ncol=6,nrow=numTypes)
cellMatrix[,1] <- initCount # count
cellMatrix[,2] <- initISa[,1] # num adv
cellMatrix[,3] <- initISa[,2] # num dis
cellMatrix[,4] <- initISa[,3] # num neu
cellMatrix[,5] <- fitness # fitness
```

```
cellMatrix[,6] <- rep(0,numTypes) # regulation timer
if(singleFounder) cellMatrix[2,5] <- 1.01    # this sets the fitness of the single founder
return(cellMatrix)
}
```

4. IS-helper.R

```
# v1.3
# 1.2 modified updateRegulator for absolute time
# 1.3 modified getMeanIS, getMeanFit and getPropIS to account for cell count

countIS <- function(cellMatrix) {
cellMatrix <- matrix(cellMatrix,ncol=6)
if(nrow(cellMatrix)<2) return(sum(cellMatrix[2:4]))
numIS <- rowSums(cellMatrix[,2:4])
return(sum(cellMatrix[numIS>0,1]))
}

compressCellMatrix <- function(cellMatrix) {
cellMatrix <- matrix(cellMatrix,ncol=6)
cellDF <- data.frame(cellMatrix)
cellDF <- ddply(cellDF,.(X2,X3,X4,X5,X6),summarize,X1=sum(X1))
cellMatrix <- matrix(c(cellDF$X1,cellDF$X2,cellDF$X3,cellDF$X4,cellDF$X5,cellDF$X6),ncol=6)
return(cellMatrix)
}

convertCellMatrix <- function(cellMatrix) {
cellMatrix <- matrix(cellMatrix,ncol=6)
oldCellMatrix <- cbind(cellMatrix[,1],rowSums(cellMatrix[,2:4]),cellMatrix[,5:6])
return(oldCellMatrix)
}

# The extinction function sums the adv, dis and neu IS columns. If the total is 0, IS are extinct in the population
extinction <- function(cellMatrix) {
cellMatrix <- matrix(cellMatrix,ncol=6)
if(nrow(cellMatrix)==1 && sum(cellMatrix[2:4])==0) return(TRUE)
else if(sum(cellMatrix[,2:4])==0) return(TRUE)
else return(FALSE)
}

# N is the sum of the count column. numIS sums the three IS columns - row by row.
# Establishment is the count of cells * an indicator whether that group of cells contains IS
establishment <- function(cellMatrix) {
cellMatrix <- matrix(cellMatrix,ncol=6)
N <- sum(cellMatrix[,1])
if(nrow(cellMatrix)==1) {
if(countIS(cellMatrix)==0) return(0)
else return(1)
}
numIS <- rowSums(cellMatrix[,2:4])
est <- sum((numIS>0)*cellMatrix[,1])
return(est/N)
}

# the cell regulator works as before.
updateRegulator <- function(cellMatrix, param, absTime=TRUE) {
cellMatrix <- matrix(cellMatrix,ncol=6)
if(param$bs == 0) return(cellMatrix)
if(nrow(cellMatrix)==1) {
if(absTime || sum(cellMatrix[2:4])>0) {
cellMatrix[6] <- cellMatrix[6]+1
return(cellMatrix)
} else {
cellMatrix <- max(cellMatrix[6]-1,0)
return(cellMatrix)
}
}
numIS <- rowSums(cellMatrix[,2:4])
if(absTime) cellMatrix[,6] <- cellMatrix[,6]+1
else {
```

```
cellMatrix[numIS>0,6] <- cellMatrix[numIS>0,6]+1
cellMatrix[numIS==0,6] <- max(cellMatrix[numIS==0,6]-1,0)
}
cellMatrix <- matrix(cellMatrix,ncol=6)
return(cellMatrix)
}

# this returns the IS count for the cell with least IS
getMinIS <- function(cellMatrix) {
cellMatrix <- matrix(cellMatrix,ncol=6)
if(nrow(cellMatrix)==1) return(sum(cellMatrix[2:4]))
vecIS <- rowSums(cellMatrix[,2:4])
minIS <- min(vecIS)
return(minIS)
}

# this returns the IS count for the cell with most IS
getMaxIS <- function(cellMatrix) {
cellMatrix <- matrix(cellMatrix,ncol=6)
if(nrow(cellMatrix)==1) return(sum(cellMatrix[2:4]))
vecIS <- rowSums(cellMatrix[,2:4])
maxIS <- max(vecIS)
return(maxIS)
}

# the mean IS count of cells - modified to account for cell count
getMeanIS <- function(cellMatrix) {
cellMatrix <- matrix(cellMatrix,ncol=6)
if(nrow(cellMatrix)==1) return(sum(cellMatrix[2:4]))
vecIS <- rep(rowSums(cellMatrix[,2:4]),cellMatrix[,1])
meanIS <- mean(vecIS)
return(meanIS)
}

# this returns the fitness for the cell with the min fitness
getMinFit <- function(cellMatrix) {
cellMatrix <- matrix(cellMatrix,ncol=6)
if(nrow(cellMatrix)==1) return(cellMatrix[5])
minFit <- min(cellMatrix[,5])
return(minFit)
}

# this returns the fitness for the cell with the max fitness
getMaxFit <- function(cellMatrix) {
cellMatrix <- matrix(cellMatrix,ncol=6)
if(nrow(cellMatrix)==1) return(cellMatrix[5])
maxFit <- max(cellMatrix[,5])
return(maxFit)
}

# this returns the mean fitness of cells - modified to account for cell count
getMeanFit <- function(cellMatrix) {
cellMatrix <- matrix(cellMatrix,ncol=6)
if(nrow(cellMatrix)==1) return(cellMatrix[5])
meanFit <- mean(rep(cellMatrix[,5],cellMatrix[,1]))
return(meanFit)
}

# this returns a vector with proportion of advantageous, deleterious and
#  neutral IS / total IS - modified to account for cell count
getPropIS <- function(cellMatrix) {
cellMatrix <- matrix(cellMatrix,ncol=6)
if(nrow(cellMatrix)==1) return(cellMatrix[2:4]/sum(cellMatrix[2:4]))
totIS <- sum(cellMatrix[,2:4]*cellMatrix[,1])
advIS <- sum(cellMatrix[,2]*cellMatrix[,1])
delIS <- sum(cellMatrix[,3]*cellMatrix[,1])
neuIS <- sum(cellMatrix[,4]*cellMatrix[,1])
propIS <- c(advIS,delIS,neuIS)/totIS
return(propIS)
}
```

5. IS-repro.R

```
# v1.00

# the population is constant during reproduction. fitness is proportional to count
reproduction <- function(cellMatrix) {
cellMatrix <- matrix(cellMatrix,ncol=6)
if(nrow(cellMatrix)==1) return(cellMatrix)
N <- sum(cellMatrix[,1])
fitness <- (cellMatrix[,1]*cellMatrix[,5])/sum(cellMatrix[,1]*cellMatrix[,5])
fitness[fitness<0]=0   # no negative probs
cellMatrix[,1] <- rmultinom(1,N,fitness)
cellMatrix <- cellMatrix[cellMatrix[,1]>0,]
cellMatrix <- matrix(cellMatrix,ncol=6)
return(cellMatrix)
}
```

6. IS-trans.R

```
# v1.03
# fixed problem with singleMutIS using an 'x' index

# calls: insertIS, exciseIS, shiftIS, extinction
transposition <- function(cellMatrix,param,fitModel="a") {
cellMatrix <- matrix(cellMatrix,ncol=6)
if(extinction(cellMatrix)) return(cellMatrix)
cellMatrix <- mutIS(cellMatrix=cellMatrix,param=param,mutType="insert",fitModel=fitModel)
cellMatrix <- mutIS(cellMatrix=cellMatrix,param=param,mutType="excise",fitModel=fitModel)
cellMatrix <- mutIS(cellMatrix=cellMatrix,param=param,mutType="shift",fitModel=fitModel)
cellMatrix <- matrix(cellMatrix,ncol=6)
return(cellMatrix)
}

singleMutIS <- function(cellMatrix,param,mutType,fitModel) { # this is code for a single row
cellMatrix <- matrix(cellMatrix,ncol=6)
numIS <- sum(cellMatrix[2:4])

if(mutType=="insert") mutProb <- param$ip
else if(mutType=="excise") mutProb <- param$ep
else if(mutType=="shift") mutProb <- param$sp
burstFactor <- param$sr+param$bs*exp(-param$br*cellMatrix[6])   # burst factor
burstProb <- param$ip*burstFactor   # burst insert prob
fitVals <- c(1+param$af,1+param$df,1) # fitness vector adv, del, neu

numMut <- rpois(1,burstProb*cellMatrix[1]*(numIS>0))
if(numMut > cellMatrix[1]) numMut = cellMatrix[1]

if(numMut>0) { # only worry mutation
mutLocs <- as.vector(rmultinom(numMut,n=1,prob=c(param$ap,param$dp,param$np))) # IS location per row (vector)
cellMatrix[1] <- cellMatrix[1]-numMut # reduce pop of mutat
newMut <- c(numMut,cellMatrix[2:6])
if(mutType=="insert") {
if(numIS <100) {
z <- sample(c(1,2,3),size=1,prob=c(param$ap,param$dp,param$np)) # z = insert location
newMut[z+1] <- newMut[z+1]+1 # insert the IS
newMut[5] <- prod(c(newMut[5],fitVals[z])) # adjust fitness
cellMatrix <- matrix(c(cellMatrix,newMut),ncol=6,byrow=TRUE)
}
} else if(mutType=="excise") {
y <- sample(c(1,2,3),size=1,prob=cellMatrix[2:4]) # prob proportional to pop
newMut[y+1] <- newMut[y+1]-1 # excise the IS
if(fitModel=="b") newMut[5] <- prod(c(newMut[5],1/fitVals[y])) # reversible model
cellMatrix <- matrix(c(cellMatrix,newMut),ncol=6,byrow=TRUE)
} else if(mutType=="shift") {
y <- sample(c(1,2,3),size=1,prob=cellMatrix[2:4])
newMut[y+1] <- newMut[y+1]-1 # remove from old location
if(fitModel=="b") newMut[5] <- prod(c(newMut[5],1/fitVals[y])) # reversible model
z <- sample(c(1,2,3),size=1,prob=c(param$ap,param$dp,param$np))
```

```
newMut[z+1] <- newMut[z+1]+1 # shift to new z location
newMut[5] <- prod(c(newMut[5],fitVals[z])) # fitness effect of shift
cellMatrix <- matrix(c(cellMatrix,newMut),ncol=6,byrow=TRUE)
}
}
cellMatrix <- matrix(cellMatrix,ncol=6) # reformat to matrix
return(cellMatrix)
}

mutIS <- function(cellMatrix,param,mutType,fitModel) {
cellMatrix <- matrix(cellMatrix,ncol=6)
if(nrow(cellMatrix)==1) return(singleMutIS(cellMatrix,param,mutType,fitModel))
else numIS <- rowSums(cellMatrix[,2:4])
if(sum(numIS)<1) {
cellMatrix <- matrix(cellMatrix,ncol=6)
return(cellMatrix)
}
if(mutType=="insert") mutProb <- param$ip
else if(mutType=="excise") mutProb <- param$ep
else if(mutType=="shift") mutProb <- param$sp
burstFactor <- param$sr+param$bs*exp(-param$br*cellMatrix[,6])   # burst factor
burstProb <- mutProb*burstFactor   # burst insert prob
fitVals <- c(1+param$af,1+param$df,1) # fitness vector adv, del, neu
numMut <- rpois(nrow(cellMatrix),burstProb*cellMatrix[,1]*(numIS>0)) # mut events per row (vec)
numMut[(cellMatrix[,1]-numMut)<0]=cellMatrix[which((cellMatrix[,1]-numMut)<0),1]
mutLocs <- t(do.call(cbind,lapply(numMut,rmultinom,n=1,prob=c(param$ap,param$dp,param$np)))) # IS location per row (matrix)
cellMatrix <- cbind(cellMatrix[,1]-rowSums(mutLocs),cellMatrix[,2:6]) # reduce pop of mutating cells
z <- 1
for(x in 1:nrow(cellMatrix)) { # x = cell type/ row index
if(numMut[x]>0) { # only worry about rows with events
newMut <- c(numMut[x],cellMatrix[x,2:6]) # copy of cell for trans event
if(mutType=="insert") {
if(numIS[x] <100) {
z <- sample(c(1,2,3),size=1,prob=c(param$ap,param$dp,param$np)) # z = insert location
newMut[z+1] <- newMut[z+1]+1 # insert the IS
newMut[5] <- prod(c(newMut[5],fitVals[z])) # adjust fitness
cellMatrix <- rbind(cellMatrix,newMut)
}
} else if(mutType=="excise") {
y <- sample(c(1,2,3),size=1,prob=cellMatrix[x,2:4]) # prob proportional to pop
newMut[y+1] <- newMut[y+1]-1 # excise the IS
if(fitModel=="b") newMut[5] <- prod(c(newMut[5],1/fitVals[y])) # reversible model
cellMatrix <- rbind(cellMatrix,newMut)
} else if(mutType=="shift") {
y <- sample(c(1,2,3),size=1,prob=cellMatrix[x,2:4])
newMut[y+1] <- newMut[y+1]-1 # remove from old location
if(fitModel=="b") newMut[5] <- prod(c(newMut[5],1/fitVals[y])) # reversible model
z <- sample(c(1,2,3),size=1,prob=c(param$ap,param$dp,param$np))
newMut[z+1] <- newMut[z+1]+1 # shift to new z location
newMut[5] <- prod(c(newMut[5],fitVals[z])) # fitness effect of shift
cellMatrix <- rbind(cellMatrix,newMut)
}
}
}
cellMatrix <- matrix(cellMatrix,ncol=6) # reformat to matrix
return(cellMatrix)
}
```
